# Supplementary material for: Comparative transcriptome and hormone analyses of roots in apple among three rootstocks with different rooting abilities
Source: PeerJ. 2024 Oct 14;12:e18244. doi: 10.7717/peerj.18244 (PMC11485133; doi:10.7717/peerj.18244)
Supplement: Supplemental Information 1 [file peerj-12-18244-s001.docx]

**Table S1** Primer sequences for the quantification of transcripts by real-time PCR

| Gene name | Primer sequence |
| --- | --- |
| *β-Actin* | F 5′-GGATTTGCTGGTGATGATGCT-3′ |
|  | R 5′-AGTTGCTCACTATGCCGTGCT-3′ |
| *MD00G1000200* | F 5′-GAAAGAAGAGGAGAAAGCCA-3′ |
|  | R 5′-GTCCCATCATTCATTGTCCT-3′ |
| *MD00G1001500* | F 5′-TGTTCATCGGCTTACTTTCA-3′ |
|  | R 5′-AACATTCGTCCCTGCTATTT-3′ |
| *MD00G1003500* | F 5′-ATACAATTCCCAACCGTACC-3′ |
|  | R 5′-TAGAGGTGGGTAAATCGAGG-3′ |
| *MD00G1007800* | F 5′-GGAGATTGCCTCTAATCCTG-3′ |
|  | R 5′-TTAGGAATGACTCCAACTGC-3′ |

**Table S2** GO functional classification of differential genes in roots of T2/HT and T2/G935

| Ontology | ID | Description | Count | Count  _Up | | | Count  _Down | | |
| --- | --- | --- | --- | --- | --- | --- | --- | --- | --- |
| Biological process | GO:0009987 | cellular process | 5262 | 2695 | | | 2567 | | |
| Biological process | GO:0008152 | metabolic process | 4450 | 2303 | | | 2147 | | |
| Biological process | GO:0050896 | response to stimulus | 2930 | 1593 | | | 1337 | | |
| Biological process | GO:0065007 | biological regulation | 2392 | 1248 | | | 1144 | | |
| Biological process | GO:0050789 | regulation of biological process | 2205 | 1159 | | | 1046 | | |
| Biological process | GO:0032502 | developmental process | 1342 | 680 | | | 662 | | |
| Biological process | GO:0032501 | multicellular organismal process | 1179 | 594 | | | 585 | | |
| Biological process | GO:0023052 | signaling | 1028 | 551 | | | 477 | | |
| Biological process | GO:0044419 | biological process involved in interspecies interaction between organisms | 998 | 586 | | | 412 | | |
| Biological process | GO:0051179 | localization | 862 | 416 | | 446 | | |  |
| Biological process | GO:0000003 | reproduction | 720 | 360 | | 360 | | |  |
| Biological process | GO:0022414 | reproductive process | 711 | 354 | | 357 | | |  |
| Biological process | GO:0048518 | positive regulation of biological process | 637 | 337 | | 300 | | |  |
| Biological process | GO:0048519 | negative regulation of biological process | 444 | 254 | | 190 | | |  |
| Biological process | GO:0040007 | growth | 305 | 157 | | 148 | | |  |
| Biological process | GO:0002376 | immune system process | 272 | 172 | | 100 | | |  |
| Biological process | GO:0048511 | rhythmic process | 86 | 50 | | 36 | | |  |
| Biological process | GO:0098754 | detoxification | 56 | 23 | | 33 | | |  |
| Biological process | GO:0040011 | locomotion | 30 | 23 | | 7 | | |  |
| Biological process | GO:0051703 | biological process involved in intraspecies interaction between organisms | 15 | 5 | | 10 | | |  |
| Biological process | GO:0016032 | viral process | 11 | 2 | | 9 | | |  |
| Biological process | GO:0043473 | pigmentation | 3 | 0 | | 3 | | |  |
| Cellular component | GO:0110165 | cellular anatomical entity | 6917 | 3505 | | 3412 | | |  |
| Cellular component | GO:0032991 | protein-containing complex | 762 | 369 | | 393 | | |  |
| Molecular function | GO:0005488 | binding | 4853 | 2616 | | 2237 | | |  |
| Molecular function | GO:0003824 | catalytic activity | 4205 | 2253 | | 1952 | | |  |
| Molecular function | GO:0140110 | transcription regulator activity | 608 | 324 | | 284 | | |  |
| Molecular function | GO:0005215 | transporter activity | 581 | 274 | | 307 | | |  |
| Molecular function | GO:0140657 | ATP-dependent activity | 313 | 159 | | 154 | | |  |
| Molecular function | GO:0060089 | molecular transducer activity | 197 | 119 | | 78 | | |  |
| Molecular function | GO:0098772 | molecular function regulator activity | 170 | 87 | | 83 | | |  |
| Molecular function | GO:0005198 | structural molecule activity | 127 | 37 | | 90 | | |  |
| Molecular function | GO:0016209 | antioxidant activity | 70 | 33 | | 37 | | |  |
| Molecular function | GO:0045182 | translation regulator activity | 49 | 28 | | 21 | | |  |
| Molecular function | GO:0003774 | cytoskeletal motor activity | 45 | 9 | | 36 | | |  |
| Molecular function | GO:0060090 | molecular adaptor activity | 28 | 15 | | 13 | | |  |
| Molecular function | GO:0044183 | protein folding chaperone | 17 | 8 | | 9 | | |  |
| Molecular function | GO:0140299 | small molecule sensor activity | 16 | 4 | | 12 | | |  |
| Molecular function | GO:0140104 | molecular carrier activity | 8 | 3 | | 5 | | |  |
| Molecular function | GO:0031386 | protein tag | 5 | 2 | | 3 | | |  |
| Molecular function | GO:0045735 | nutrient reservoir activity | 4 | 3 | | 1 | | |  |
| Molecular function | GO:0090729 | toxin activity | 4 | 4 | | 0 | | |  |
| Molecular function | GO:0140776 | protein-containing complex destabilizing activity | 2 | | 2 | | | 0 | |

**Table S3** GO functional classification of differential genes in roots of T2/HT and T2/J2

| Ontology | ID | Description | Count | Count  _Up | | | Count  _Down | | |
| --- | --- | --- | --- | --- | --- | --- | --- | --- | --- |
| Biological process | GO:0009987 | cellular process | 5397 | 2461 | | | 2936 | | |
| Biological process | GO:0008152 | metabolic process | 4617 | 2165 | | | 2452 | | |
| Biological process | GO:0050896 | response to stimulus | 3045 | 1413 | | | 1632 | | |
| Biological process | GO:0065007 | biological regulation | 2441 | 1077 | | | 1364 | | |
| Biological process | GO:0050789 | regulation of biological process | 2247 | 1005 | | | 1242 | | |
| Biological process | GO:0032502 | developmental process | 1373 | 590 | | | 783 | | |
| Biological process | GO:0032501 | multicellular organismal process | 1191 | 508 | | | 683 | | |
| Biological process | GO:0023052 | signaling | 1049 | 465 | | | 584 | | |
| Biological process | GO:0044419 | biological process involved in interspecies interaction between organisms | 1008 | 447 | | | 561 | | |
| Biological process | GO:0051179 | localization | 946 | 412 | | 534 | | |  |
| Biological process | GO:0000003 | reproduction | 698 | 280 | | 418 | | |  |
| Biological process | GO:0022414 | reproductive process | 693 | 276 | | 417 | | |  |
| Biological process | GO:0048518 | positive regulation of biological process | 591 | 260 | | 331 | | |  |
| Biological process | GO:0048519 | negative regulation of biological process | 441 | 202 | | 239 | | |  |
| Biological process | GO:0040007 | growth | 320 | 136 | | 184 | | |  |
| Biological process | GO:0002376 | immune system process | 236 | 100 | | 136 | | |  |
| Biological process | GO:0048511 | rhythmic process | 77 | 37 | | 40 | | |  |
| Biological process | GO:0098754 | detoxification | 60 | 24 | | 36 | | |  |
| Biological process | GO:0040011 | locomotion | 24 | 14 | | 10 | | |  |
| Biological process | GO:0051703 | biological process involved in intraspecies interaction between organisms | 17 | 7 | | 10 | | |  |
| Biological process | GO:0016032 | viral process | 15 | 6 | | 9 | | |  |
| Biological process | GO:0043473 | pigmentation | 3 | 1 | | 2 | | |  |
| Cellular component | GO:0110165 | cellular anatomical entity | 7251 | 3364 | | 3887 | | |  |
| Cellular component | GO:0032991 | protein-containing complex | 761 | 354 | | 407 | | |  |
| Molecular function | GO:0005488 | binding | 5056 | 2354 | | 2702 | | |  |
| Molecular function | GO:0003824 | catalytic activity | 4423 | 2078 | | 2345 | | |  |
| Molecular function | GO:0005215 | transporter activity | 651 | 266 | | 385 | | |  |
| Molecular function | GO:0140110 | transcription regulator activity | 637 | 300 | | 337 | | |  |
| Molecular function | GO:0140657 | ATP-dependent activity | 310 | 106 | | 204 | | |  |
| Molecular function | GO:0060089 | molecular transducer activity | 201 | 93 | | 108 | | |  |
| Molecular function | GO:0098772 | molecular function regulator activity | 185 | 90 | | 95 | | |  |
| Molecular function | GO:0005198 | structural molecule activity | 118 | 58 | | 60 | | |  |
| Molecular function | GO:0016209 | antioxidant activity | 75 | 42 | | 33 | | |  |
| Molecular function | GO:0003774 | cytoskeletal motor activity | 36 | 10 | | 26 | | |  |
| Molecular function | GO:0045182 | translation regulator activity | 34 | 9 | | 25 | | |  |
| Molecular function | GO:0140299 | small molecule sensor activity | 25 | 5 | | 20 | | |  |
| Molecular function | GO:0060090 | molecular adaptor activity | 24 | 11 | | 13 | | |  |
| Molecular function | GO:0044183 | protein folding chaperone | 21 | 10 | | 11 | | |  |
| Molecular function | GO:0140104 | molecular carrier activity | 17 | 9 | | 8 | | |  |
| Molecular function | GO:0045735 | nutrient reservoir activity | 9 | 6 | | 3 | | |  |
| Molecular function | GO:0031386 | protein tag | 7 | 5 | | 2 | | |  |
| Molecular function | GO:0090729 | toxin activity | 2 | 2 | | 0 | | |  |
| Molecular function | GO:0140776 | protein-containing complex destabilizing activity | 2 | | 1 | | | 1 | |

**Table S4** GO functional classification of differential genes in roots of T2/J2 and T2/G935

| Ontology | ID | Description | Count | Count  _Up | | | Count  _Down | | |
| --- | --- | --- | --- | --- | --- | --- | --- | --- | --- |
| Biological process | GO:0009987 | cellular process | 4361 | 2209 | | | 2152 | | |
| Biological process | GO:0008152 | metabolic process | 3619 | 1816 | | | 1803 | | |
| Biological process | GO:0050896 | response to stimulus | 2499 | 1362 | | | 1137 | | |
| Biological process | GO:0065007 | biological regulation | 1920 | 1033 | | | 887 | | |
| Biological process | GO:0050789 | regulation of biological process | 1766 | 944 | | | 822 | | |
| Biological process | GO:0032502 | developmental process | 989 | 533 | | | 456 | | |
| Biological process | GO:0044419 | biological process involved in interspecies interaction between organisms | 954 | 588 | | | 366 | | |
| Biological process | GO:0023052 | signaling | 895 | 497 | | | 398 | | |
| Biological process | GO:0032501 | multicellular organismal process | 873 | 475 | | | 398 | | |
| Biological process | GO:0051179 | localization | 710 | 331 | | | 379 | | |
| Biological process | GO:0000003 | reproduction | 521 | 295 | | 226 | | |  |
| Biological process | GO:0022414 | reproductive process | 517 | 293 | | 224 | | |  |
| Biological process | GO:0048518 | positive regulation of biological process | 503 | 275 | | 228 | | |  |
| Biological process | GO:0048519 | negative regulation of biological process | 353 | 191 | | 162 | | |  |
| Biological process | GO:0002376 | immune system process | 250 | 165 | | 85 | | |  |
| Biological process | GO:0040007 | growth | 228 | 131 | | 97 | | |  |
| Biological process | GO:0048511 | rhythmic process | 54 | 32 | | 22 | | |  |
| Biological process | GO:0098754 | detoxification | 35 | 17 | | 18 | | |  |
| Biological process | GO:0040011 | locomotion | 28 | 19 | | 9 | | |  |
| Biological process | GO:0051703 | biological process involved in intraspecies interaction between organisms | 18 | 13 | | 5 | | |  |
| Biological process | GO:0016032 | viral process | 11 | 5 | | 6 | | |  |
| Biological process | GO:0043473 | pigmentation | 2 | 1 | | 1 | | |  |
| Cellular component | GO:0110165 | cellular anatomical entity | 5792 | 2829 | | 2963 | | |  |
| Cellular component | GO:0032991 | protein-containing complex | 682 | 261 | | 421 | | |  |
| Molecular function | GO:0005488 | binding | 4094 | 2172 | | 1922 | | |  |
| Molecular function | GO:0003824 | catalytic activity | 3429 | 1843 | | 1586 | | |  |
| Molecular function | GO:0005215 | transporter activity | 465 | 239 | | 226 | | |  |
| Molecular function | GO:0140110 | transcription regulator activity | 465 | 240 | | 225 | | |  |
| Molecular function | GO:0140657 | ATP-dependent activity | 229 | 145 | | 84 | | |  |
| Molecular function | GO:0060089 | molecular transducer activity | 172 | 107 | | 65 | | |  |
| Molecular function | GO:0098772 | molecular function regulator activity | 157 | 64 | | 93 | | |  |
| Molecular function | GO:0005198 | structural molecule activity | 155 | 29 | | 126 | | |  |
| Molecular function | GO:0016209 | antioxidant activity | 66 | 25 | | 41 | | |  |
| Molecular function | GO:0045182 | translation regulator activity | 28 | 21 | | 7 | | |  |
| Molecular function | GO:0060090 | molecular adaptor activity | 21 | 7 | | 14 | | |  |
| Molecular function | GO:0044183 | protein folding chaperone | 18 | 8 | | 10 | | |  |
| Molecular function | GO:0140104 | molecular carrier activity | 18 | 8 | | 10 | | |  |
| Molecular function | GO:0003774 | cytoskeletal motor activity | 15 | 6 | | 9 | | |  |
| Molecular function | GO:0140299 | small molecule sensor activity | 12 | 8 | | 4 | | |  |
| Molecular function | GO:0031386 | protein tag | 10 | 1 | | 9 | | |  |
| Molecular function | GO:0045735 | nutrient reservoir activity | 9 | 3 | | 6 | | |  |
| Molecular function | GO:0090729 | toxin activity | 2 | 2 | | 0 | | |  |
| Molecular function | GO:0140776 | protein-containing complex destabilizing activity | 2 | 2 | | 0 | | |  |
| Molecular function | GO:0140223 | general transcription initiation factor activity | 1 | | 0 | | | 1 | |

**Table S5** Statistics of the number of DEGs corresponding to KEGG pathways of T2/HT and T2/G935

| ID | Description | Count | Up | Down |
| --- | --- | --- | --- | --- |
| ko04626 | Plant-pathogen interaction | 777 | 480 | 297 |
| ko04016 | MAPK signaling pathway - plant | 302 | 183 | 119 |
| ko00941 | Flavonoid biosynthesis | 80 | 36 | 44 |
| ko04075 | Plant hormone signal transduction | 378 | 187 | 191 |
| ko01110 | Biosynthesis of secondary metabolites | 850 | 440 | 410 |
| ko00100 | Steroid biosynthesis | 43 | 20 | 23 |
| ko00430 | Taurine and hypotaurine metabolism | 15 | 10 | 5 |
| ko00561 | Glycerolipid metabolism | 49 | 33 | 16 |
| ko00564 | Glycerophospholipid metabolism | 63 | 46 | 17 |
| ko00900 | Terpenoid backbone biosynthesis | 34 | 23 | 11 |
| ko04070 | Phosphatidylinositol signaling system | 45 | 26 | 19 |
| ko00905 | Brassinosteroid biosynthesis | 29 | 12 | 17 |
| ko00053 | Ascorbate and aldarate metabolism | 45 | 23 | 22 |
| ko00945 | Stilbenoid, diarylheptanoid and gingerol biosynthesis | 36 | 22 | 14 |
| ko00592 | alpha-Linolenic acid metabolism | 56 | 36 | 20 |
| ko00562 | Inositol phosphate metabolism | 49 | 31 | 18 |
| ko00790 | Folate biosynthesis | 14 | 9 | 5 |
| ko00906 | Carotenoid biosynthesis | 32 | 15 | 17 |
| ko00565 | Ether lipid metabolism | 16 | 10 | 6 |
| ko00944 | Flavone and flavonol biosynthesis | 15 | 4 | 11 |
| ko00904 | Diterpenoid biosynthesis | 24 | 12 | 12 |
| ko00940 | Phenylpropanoid biosynthesis | 124 | 65 | 59 |
| ko00908 | Zeatin biosynthesis | 11 | 9 | 2 |
| ko00942 | Anthocyanin biosynthesis | 4 | 0 | 4 |
| ko00650 | Butanoate metabolism | 13 | 9 | 4 |
| ko00909 | Sesquiterpenoid and triterpenoid biosynthesis | 22 | 17 | 5 |
| ko00902 | Monoterpenoid biosynthesis | 7 | 2 | 5 |
| ko00531 | Glycosaminoglycan degradation | 14 | 4 | 10 |
| ko00040 | Pentose and glucuronate interconversions | 78 | 29 | 49 |
| ko00999 | Biosynthesis of various plant secondary metabolites | 65 | 30 | 35 |
| ko00604 | Glycosphingolipid biosynthesis - ganglio series | 9 | 2 | 7 |
| ko00511 | Other glycan degradation | 15 | 4 | 11 |
| ko00052 | Galactose metabolism | 35 | 19 | 16 |
| ko00480 | Glutathione metabolism | 54 | 24 | 30 |
| ko03410 | Base excision repair | 24 | 10 | 14 |
| ko00260 | Glycine, serine and threonine metabolism | 35 | 13 | 22 |
| ko00591 | Linoleic acid metabolism | 12 | 9 | 3 |
| ko00410 | beta-Alanine metabolism | 29 | 13 | 16 |
| ko03430 | Mismatch repair | 21 | 8 | 13 |
| ko01100 | Metabolic pathways | 1303 | 642 | 661 |
| ko00051 | Fructose and mannose metabolism | 33 | 13 | 20 |
| ko00350 | Tyrosine metabolism | 26 | 17 | 9 |
| ko00910 | Nitrogen metabolism | 18 | 6 | 12 |
| ko00750 | Vitamin B6 metabolism | 12 | 7 | 5 |
| ko00500 | Starch and sucrose metabolism | 106 | 40 | 66 |
| ko00670 | One carbon pool by folate | 10 | 3 | 7 |
| ko00960 | Tropane, piperidine and pyridine alkaloid biosynthesis | 16 | 6 | 10 |
| ko00514 | Other types of O-glycan biosynthesis | 8 | 5 | 3 |
| ko04814 | Motor proteins | 56 | 9 | 47 |
| ko00620 | Pyruvate metabolism | 56 | 39 | 17 |
| ko00860 | Porphyrin metabolism | 24 | 5 | 19 |
| ko00520 | Amino sugar and nucleotide sugar metabolism | 62 | 23 | 39 |
| ko04712 | Circadian rhythm - plant | 48 | 19 | 29 |
| ko03030 | DNA replication | 26 | 8 | 18 |
| ko02010 | ABC transporters | 48 | 21 | 27 |
| ko00071 | Fatty acid degradation | 28 | 16 | 12 |
| ko00130 | Ubiquinone and other terpenoid-quinone biosynthesis | 22 | 8 | 14 |
| ko00195 | Photosynthesis | 24 | 13 | 11 |
| ko00250 | Alanine, aspartate and glutamate metabolism | 26 | 7 | 19 |
| ko00380 | Tryptophan metabolism | 46 | 21 | 25 |
| ko00261 | Monobactam biosynthesis | 5 | 4 | 1 |
| ko00640 | Propanoate metabolism | 20 | 6 | 14 |
| ko00590 | Arachidonic acid metabolism | 6 | 4 | 2 |
| ko00460 | Cyanoamino acid metabolism | 37 | 16 | 21 |
| ko00360 | Phenylalanine metabolism | 16 | 8 | 8 |
| ko00966 | Glucosinolate biosynthesis | 6 | 2 | 4 |
| ko00600 | Sphingolipid metabolism | 20 | 7 | 13 |
| ko01240 | Biosynthesis of cofactors | 113 | 55 | 58 |
| ko00920 | Sulfur metabolism | 14 | 9 | 5 |
| ko00010 | Glycolysis / Gluconeogenesis | 61 | 44 | 17 |
| ko00660 | C5-Branched dibasic acid metabolism | 3 | 3 | 0 |
| ko00965 | Betalain biosynthesis | 2 | 1 | 1 |
| ko00310 | Lysine degradation | 23 | 14 | 9 |
| ko00760 | Nicotinate and nicotinamide metabolism | 8 | 7 | 1 |
| ko00770 | Pantothenate and CoA biosynthesis | 15 | 11 | 4 |
| ko00290 | Valine, leucine and isoleucine biosynthesis | 7 | 5 | 2 |
| ko01250 | Biosynthesis of nucleotide sugars | 37 | 18 | 19 |
| ko00270 | Cysteine and methionine metabolism | 49 | 24 | 25 |
| ko00780 | Biotin metabolism | 8 | 4 | 4 |
| ko03420 | Nucleotide excision repair | 32 | 14 | 18 |
| ko00603 | Glycosphingolipid biosynthesis - globo and isoglobo series | 4 | 1 | 3 |
| ko00950 | Isoquinoline alkaloid biosynthesis | 10 | 6 | 4 |
| ko04130 | SNARE interactions in vesicular transport | 16 | 12 | 4 |
| ko00330 | Arginine and proline metabolism | 27 | 11 | 16 |
| ko00601 | Glycosphingolipid biosynthesis - lacto and neolacto series | 1 | 1 | 0 |
| ko00300 | Lysine biosynthesis | 5 | 3 | 2 |
| ko03250 | Viral life cycle - HIV-1 | 13 | 9 | 4 |
| ko00030 | Pentose phosphate pathway | 21 | 15 | 6 |
| ko00280 | Valine, leucine and isoleucine degradation | 23 | 8 | 15 |
| ko00020 | Citrate cycle (TCA cycle) | 26 | 17 | 9 |
| ko04136 | Autophagy - other | 18 | 9 | 9 |
| ko00730 | Thiamine metabolism | 8 | 4 | 4 |
| ko01210 | 2-Oxocarboxylic acid metabolism | 22 | 12 | 10 |
| ko00073 | Cutin, suberine and wax biosynthesis | 19 | 9 | 10 |
| ko01230 | Biosynthesis of amino acids | 96 | 50 | 46 |
| ko03020 | RNA polymerase | 20 | 3 | 17 |
| ko03060 | Protein export | 32 | 29 | 3 |
| ko00543 | Exopolysaccharide biosynthesis | 1 | 1 | 0 |
| ko00061 | Fatty acid biosynthesis | 17 | 8 | 9 |
| ko04122 | Sulfur relay system | 2 | 2 | 0 |
| ko03440 | Homologous recombination | 20 | 5 | 15 |
| ko04146 | Peroxisome | 47 | 31 | 16 |
| ko00220 | Arginine biosynthesis | 15 | 3 | 12 |
| ko01212 | Fatty acid metabolism | 27 | 14 | 13 |
| ko00440 | Phosphonate and phosphinate metabolism | 1 | 1 | 0 |
| ko03450 | Non-homologous end-joining | 1 | 0 | 1 |
| ko01040 | Biosynthesis of unsaturated fatty acids | 8 | 4 | 4 |
| ko00630 | Glyoxylate and dicarboxylate metabolism | 25 | 11 | 14 |
| ko01200 | Carbon metabolism | 114 | 64 | 50 |
| ko00340 | Histidine metabolism | 4 | 0 | 4 |
| ko00400 | Phenylalanine, tyrosine and tryptophan biosynthesis | 14 | 6 | 8 |
| ko00470 | D-Amino acid metabolism | 1 | 0 | 1 |
| ko00943 | Isoflavonoid biosynthesis | 1 | 1 | 0 |
| ko00240 | Pyrimidine metabolism | 19 | 12 | 7 |
| ko00785 | Lipoic acid metabolism | 10 | 3 | 7 |
| ko03083 | Polycomb repressive complex | 31 | 18 | 13 |
| ko00740 | Riboflavin metabolism | 5 | 1 | 4 |
| ko04144 | Endocytosis | 77 | 43 | 34 |
| ko00710 | Carbon fixation in photosynthetic organisms | 26 | 19 | 7 |
| ko00196 | Photosynthesis - antenna proteins | 3 | 1 | 2 |
| ko03082 | ATP-dependent chromatin remodeling | 27 | 20 | 7 |
| ko04145 | Phagosome | 23 | 7 | 16 |
| ko00450 | Selenocompound metabolism | 3 | 3 | 0 |
| ko03018 | RNA degradation | 45 | 21 | 24 |
| ko03022 | Basal transcription factors | 13 | 7 | 6 |
| ko03015 | mRNA surveillance pathway | 51 | 33 | 18 |
| ko03008 | Ribosome biogenesis in eukaryotes | 22 | 11 | 11 |
| ko00563 | Glycosylphosphatidylinositol (GPI)-anchor biosynthesis | 4 | 0 | 4 |
| ko00970 | Aminoacyl-tRNA biosynthesis | 21 | 13 | 8 |
| ko00230 | Purine metabolism | 26 | 14 | 12 |
| ko01232 | Nucleotide metabolism | 20 | 12 | 8 |
| ko00062 | Fatty acid elongation | 6 | 3 | 3 |
| ko03013 | Nucleocytoplasmic transport | 38 | 21 | 17 |
| ko00510 | N-Glycan biosynthesis | 9 | 3 | 6 |
| ko04120 | Ubiquitin mediated proteolysis | 47 | 30 | 17 |
| ko00190 | Oxidative phosphorylation | 39 | 20 | 19 |
| ko03040 | Spliceosome | 84 | 57 | 27 |
| ko00513 | Various types of N-glycan biosynthesis | 3 | 2 | 1 |
| ko04141 | Protein processing in endoplasmic reticulum | 79 | 47 | 32 |
| ko03050 | Proteasome | 6 | 0 | 6 |
| ko03010 | Ribosome | 71 | 19 | 52 |

**Table S6** Statistics of the number of DEGs corresponding to KEGG pathways of T2/HT and T2/J2

| ID | Description | Count | Up | Down |
| --- | --- | --- | --- | --- |
| ko04626 | Plant-pathogen interaction | 767 | 328 | 439 |
| ko04075 | Plant hormone signal transduction | 453 | 194 | 259 |
| ko01110 | Biosynthesis of secondary metabolites | 920 | 432 | 488 |
| ko00910 | Nitrogen metabolism | 36 | 17 | 19 |
| ko04016 | MAPK signaling pathway - plant | 291 | 153 | 138 |
| ko00941 | Flavonoid biosynthesis | 80 | 41 | 39 |
| ko01100 | Metabolic pathways | 1444 | 648 | 796 |
| ko00945 | Stilbenoid, diarylheptanoid and gingerol biosynthesis | 42 | 26 | 16 |
| ko00052 | Galactose metabolism | 47 | 20 | 27 |
| ko00100 | Steroid biosynthesis | 39 | 9 | 30 |
| ko00900 | Terpenoid backbone biosynthesis | 36 | 24 | 12 |
| ko00940 | Phenylpropanoid biosynthesis | 141 | 82 | 59 |
| ko00480 | Glutathione metabolism | 66 | 37 | 29 |
| ko03430 | Mismatch repair | 28 | 11 | 17 |
| ko00053 | Ascorbate and aldarate metabolism | 47 | 21 | 26 |
| ko00500 | Starch and sucrose metabolism | 125 | 33 | 92 |
| ko00780 | Biotin metabolism | 16 | 8 | 8 |
| ko00905 | Brassinosteroid biosynthesis | 29 | 10 | 19 |
| ko00904 | Diterpenoid biosynthesis | 27 | 11 | 16 |
| ko03030 | DNA replication | 35 | 11 | 24 |
| ko00591 | Linoleic acid metabolism | 16 | 8 | 8 |
| ko00360 | Phenylalanine metabolism | 24 | 14 | 10 |
| ko00592 | alpha-Linolenic acid metabolism | 57 | 27 | 30 |
| ko00860 | Porphyrin metabolism | 31 | 6 | 25 |
| ko00999 | Biosynthesis of various plant secondary metabolites | 73 | 30 | 43 |
| ko00531 | Glycosaminoglycan degradation | 16 | 4 | 12 |
| ko00620 | Pyruvate metabolism | 67 | 28 | 39 |
| ko00906 | Carotenoid biosynthesis | 33 | 17 | 16 |
| ko00909 | Sesquiterpenoid and triterpenoid biosynthesis | 24 | 12 | 12 |
| ko00520 | Amino sugar and nucleotide sugar metabolism | 73 | 31 | 42 |
| ko00250 | Alanine, aspartate and glutamate metabolism | 33 | 12 | 21 |
| ko00040 | Pentose and glucuronate interconversions | 84 | 30 | 54 |
| ko00010 | Glycolysis / Gluconeogenesis | 76 | 40 | 36 |
| ko03410 | Base excision repair | 27 | 10 | 17 |
| ko00561 | Glycerolipid metabolism | 41 | 23 | 18 |
| ko00430 | Taurine and hypotaurine metabolism | 10 | 7 | 3 |
| ko00960 | Tropane, piperidine and pyridine alkaloid biosynthesis | 19 | 10 | 9 |
| ko00061 | Fatty acid biosynthesis | 28 | 15 | 13 |
| ko00460 | Cyanoamino acid metabolism | 45 | 22 | 23 |
| ko00130 | Ubiquinone and other terpenoid-quinone biosynthesis | 27 | 10 | 17 |
| ko01240 | Biosynthesis of cofactors | 132 | 66 | 66 |
| ko01250 | Biosynthesis of nucleotide sugars | 48 | 20 | 28 |
| ko00511 | Other glycan degradation | 16 | 4 | 12 |
| ko00071 | Fatty acid degradation | 33 | 15 | 18 |
| ko00220 | Arginine biosynthesis | 25 | 12 | 13 |
| ko02010 | ABC transporters | 54 | 19 | 35 |
| ko00590 | Arachidonic acid metabolism | 8 | 5 | 3 |
| ko00350 | Tyrosine metabolism | 28 | 18 | 10 |
| ko00604 | Glycosphingolipid biosynthesis - ganglio series | 9 | 3 | 6 |
| ko00196 | Photosynthesis - antenna proteins | 10 | 0 | 10 |
| ko00740 | Riboflavin metabolism | 13 | 7 | 6 |
| ko00340 | Histidine metabolism | 10 | 1 | 9 |
| ko00310 | Lysine degradation | 29 | 9 | 20 |
| ko00564 | Glycerophospholipid metabolism | 53 | 35 | 18 |
| ko00232 | Caffeine metabolism | 2 | 1 | 1 |
| ko00051 | Fructose and mannose metabolism | 34 | 17 | 17 |
| ko00942 | Anthocyanin biosynthesis | 3 | 0 | 3 |
| ko03440 | Homologous recombination | 29 | 9 | 20 |
| ko00908 | Zeatin biosynthesis | 9 | 7 | 2 |
| ko00380 | Tryptophan metabolism | 50 | 25 | 25 |
| ko00943 | Isoflavonoid biosynthesis | 4 | 2 | 2 |
| ko00330 | Arginine and proline metabolism | 34 | 10 | 24 |
| ko00260 | Glycine, serine and threonine metabolism | 34 | 11 | 23 |
| ko00790 | Folate biosynthesis | 10 | 5 | 5 |
| ko00601 | Glycosphingolipid biosynthesis - lacto and neolacto series | 2 | 1 | 1 |
| ko00565 | Ether lipid metabolism | 12 | 7 | 5 |
| ko00902 | Monoterpenoid biosynthesis | 5 | 1 | 4 |
| ko00400 | Phenylalanine, tyrosine and tryptophan biosynthesis | 22 | 12 | 10 |
| ko00950 | Isoquinoline alkaloid biosynthesis | 13 | 10 | 3 |
| ko00600 | Sphingolipid metabolism | 22 | 9 | 13 |
| ko00670 | One carbon pool by folate | 9 | 1 | 8 |
| ko03060 | Protein export | 40 | 21 | 19 |
| ko00944 | Flavone and flavonol biosynthesis | 11 | 4 | 7 |
| ko01212 | Fatty acid metabolism | 36 | 17 | 19 |
| ko00261 | Monobactam biosynthesis | 5 | 2 | 3 |
| ko00730 | Thiamine metabolism | 11 | 5 | 6 |
| ko00630 | Glyoxylate and dicarboxylate metabolism | 34 | 13 | 21 |
| ko00603 | Glycosphingolipid biosynthesis - globo and isoglobo series | 5 | 2 | 3 |
| ko00543 | Exopolysaccharide biosynthesis | 2 | 2 | 0 |
| ko00750 | Vitamin B6 metabolism | 10 | 8 | 2 |
| ko00470 | D-Amino acid metabolism | 3 | 2 | 1 |
| ko00650 | Butanoate metabolism | 9 | 5 | 4 |
| ko00195 | Photosynthesis | 23 | 7 | 16 |
| ko04712 | Circadian rhythm - plant | 46 | 16 | 30 |
| ko00770 | Pantothenate and CoA biosynthesis | 16 | 10 | 6 |
| ko00965 | Betalain biosynthesis | 2 | 0 | 2 |
| ko00514 | Other types of O-glycan biosynthesis | 6 | 2 | 4 |
| ko00410 | beta-Alanine metabolism | 24 | 5 | 19 |
| ko04136 | Autophagy - other | 20 | 14 | 6 |
| ko03450 | Non-homologous end-joining | 2 | 1 | 1 |
| ko04146 | Peroxisome | 53 | 31 | 22 |
| ko00280 | Valine, leucine and isoleucine degradation | 24 | 6 | 18 |
| ko00785 | Lipoic acid metabolism | 14 | 7 | 7 |
| ko03018 | RNA degradation | 59 | 30 | 29 |
| ko00270 | Cysteine and methionine metabolism | 49 | 23 | 26 |
| ko04130 | SNARE interactions in vesicular transport | 16 | 10 | 6 |
| ko00710 | Carbon fixation in photosynthetic organisms | 33 | 16 | 17 |
| ko01232 | Nucleotide metabolism | 31 | 16 | 15 |
| ko00920 | Sulfur metabolism | 12 | 6 | 6 |
| ko00030 | Pentose phosphate pathway | 21 | 11 | 10 |
| ko03420 | Nucleotide excision repair | 31 | 13 | 18 |
| ko00230 | Purine metabolism | 38 | 12 | 26 |
| ko04814 | Motor proteins | 48 | 10 | 38 |
| ko00640 | Propanoate metabolism | 16 | 2 | 14 |
| ko00966 | Glucosinolate biosynthesis | 4 | 1 | 3 |
| ko00450 | Selenocompound metabolism | 6 | 1 | 5 |
| ko00073 | Cutin, suberine and wax biosynthesis | 19 | 12 | 7 |
| ko01200 | Carbon metabolism | 124 | 54 | 70 |
| ko01210 | 2-Oxocarboxylic acid metabolism | 22 | 13 | 9 |
| ko04070 | Phosphatidylinositol signaling system | 27 | 12 | 15 |
| ko00300 | Lysine biosynthesis | 4 | 3 | 1 |
| ko00240 | Pyrimidine metabolism | 22 | 10 | 12 |
| ko00020 | Citrate cycle (TCA cycle) | 24 | 17 | 7 |
| ko00562 | Inositol phosphate metabolism | 31 | 14 | 17 |
| ko03020 | RNA polymerase | 18 | 6 | 12 |
| ko00660 | C5-Branched dibasic acid metabolism | 1 | 1 | 0 |
| ko01230 | Biosynthesis of amino acids | 94 | 52 | 42 |
| ko04122 | Sulfur relay system | 1 | 1 | 0 |
| ko03083 | Polycomb repressive complex | 32 | 17 | 15 |
| ko01040 | Biosynthesis of unsaturated fatty acids | 7 | 1 | 6 |
| ko00760 | Nicotinate and nicotinamide metabolism | 4 | 2 | 2 |
| ko04145 | Phagosome | 24 | 4 | 20 |
| ko00290 | Valine, leucine and isoleucine biosynthesis | 3 | 2 | 1 |
| ko00970 | Aminoacyl-tRNA biosynthesis | 25 | 3 | 22 |
| ko00190 | Oxidative phosphorylation | 51 | 39 | 12 |
| ko03022 | Basal transcription factors | 15 | 7 | 8 |
| ko03250 | Viral life cycle - HIV-1 | 8 | 4 | 4 |
| ko00563 | Glycosylphosphatidylinositol (GPI)-anchor biosynthesis | 5 | 2 | 3 |
| ko00510 | N-Glycan biosynthesis | 13 | 8 | 5 |
| ko03008 | Ribosome biogenesis in eukaryotes | 24 | 16 | 8 |
| ko03082 | ATP-dependent chromatin remodeling | 25 | 18 | 7 |
| ko00062 | Fatty acid elongation | 7 | 2 | 5 |
| ko00513 | Various types of N-glycan biosynthesis | 7 | 4 | 3 |
| ko03050 | Proteasome | 14 | 7 | 7 |
| ko03013 | Nucleocytoplasmic transport | 40 | 17 | 23 |
| ko04120 | Ubiquitin mediated proteolysis | 49 | 31 | 18 |
| ko04141 | Protein processing in endoplasmic reticulum | 92 | 71 | 21 |
| ko04144 | Endocytosis | 63 | 37 | 26 |
| ko03015 | mRNA surveillance pathway | 39 | 15 | 24 |
| ko03040 | Spliceosome | 76 | 49 | 27 |
| ko03010 | Ribosome | 59 | 38 | 21 |

**Table S7** Statistics of the number of DEGs corresponding to KEGG pathways of T2/J2 and T2/G935

| ID | Description | Count | Up | Down |
| --- | --- | --- | --- | --- |
| ko04626 | Plant-pathogen interaction | 741 | 510 | 231 |
| ko04016 | MAPK signaling pathway - plant | 243 | 143 | 100 |
| ko00945 | Stilbenoid, diarylheptanoid and gingerol biosynthesis | 36 | 15 | 21 |
| ko00910 | Nitrogen metabolism | 24 | 11 | 13 |
| ko00430 | Taurine and hypotaurine metabolism | 12 | 7 | 5 |
| ko04075 | Plant hormone signal transduction | 303 | 177 | 126 |
| ko00941 | Flavonoid biosynthesis | 59 | 24 | 35 |
| ko03060 | Protein export | 45 | 29 | 16 |
| ko00480 | Glutathione metabolism | 56 | 16 | 40 |
| ko00053 | Ascorbate and aldarate metabolism | 38 | 22 | 16 |
| ko00940 | Phenylpropanoid biosynthesis | 111 | 49 | 62 |
| ko00906 | Carotenoid biosynthesis | 29 | 9 | 20 |
| ko00909 | Sesquiterpenoid and triterpenoid biosynthesis | 21 | 11 | 10 |
| ko00591 | Linoleic acid metabolism | 13 | 10 | 3 |
| ko00561 | Glycerolipid metabolism | 36 | 18 | 18 |
| ko00310 | Lysine degradation | 28 | 19 | 9 |
| ko00380 | Tryptophan metabolism | 47 | 21 | 26 |
| ko00905 | Brassinosteroid biosynthesis | 22 | 10 | 12 |
| ko00943 | Isoflavonoid biosynthesis | 5 | 2 | 3 |
| ko00592 | alpha-Linolenic acid metabolism | 44 | 28 | 16 |
| ko00010 | Glycolysis / Gluconeogenesis | 62 | 36 | 26 |
| ko00780 | Biotin metabolism | 11 | 4 | 7 |
| ko00130 | Ubiquinone and other terpenoid-quinone biosynthesis | 22 | 11 | 11 |
| ko03430 | Mismatch repair | 19 | 10 | 9 |
| ko00520 | Amino sugar and nucleotide sugar metabolism | 57 | 25 | 32 |
| ko00920 | Sulfur metabolism | 16 | 9 | 7 |
| ko00190 | Oxidative phosphorylation | 62 | 11 | 51 |
| ko00564 | Glycerophospholipid metabolism | 45 | 26 | 19 |
| ko03410 | Base excision repair | 21 | 10 | 11 |
| ko00052 | Galactose metabolism | 30 | 16 | 14 |
| ko00750 | Vitamin B6 metabolism | 11 | 2 | 9 |
| ko00904 | Diterpenoid biosynthesis | 18 | 8 | 10 |
| ko00360 | Phenylalanine metabolism | 16 | 6 | 10 |
| ko00908 | Zeatin biosynthesis | 8 | 5 | 3 |
| ko00902 | Monoterpenoid biosynthesis | 5 | 2 | 3 |
| ko00051 | Fructose and mannose metabolism | 28 | 12 | 16 |
| ko00604 | Glycosphingolipid biosynthesis - ganglio series | 7 | 2 | 5 |
| ko00790 | Folate biosynthesis | 9 | 8 | 1 |
| ko00740 | Riboflavin metabolism | 10 | 4 | 6 |
| ko00531 | Glycosaminoglycan degradation | 10 | 5 | 5 |
| ko02010 | ABC transporters | 41 | 23 | 18 |
| ko01110 | Biosynthesis of secondary metabolites | 655 | 333 | 322 |
| ko00603 | Glycosphingolipid biosynthesis - globo and isoglobo series | 5 | 1 | 4 |
| ko00620 | Pyruvate metabolism | 47 | 34 | 13 |
| ko00562 | Inositol phosphate metabolism | 34 | 22 | 12 |
| ko00400 | Phenylalanine, tyrosine and tryptophan biosynthesis | 18 | 8 | 10 |
| ko00061 | Fatty acid biosynthesis | 19 | 9 | 10 |
| ko00350 | Tyrosine metabolism | 20 | 9 | 11 |
| ko00543 | Exopolysaccharide biosynthesis | 2 | 1 | 1 |
| ko00942 | Anthocyanin biosynthesis | 2 | 1 | 1 |
| ko00071 | Fatty acid degradation | 23 | 15 | 8 |
| ko00860 | Porphyrin metabolism | 19 | 11 | 8 |
| ko00261 | Monobactam biosynthesis | 4 | 3 | 1 |
| ko00999 | Biosynthesis of various plant secondary metabolites | 49 | 24 | 25 |
| ko00511 | Other glycan degradation | 10 | 3 | 7 |
| ko00563 | Glycosylphosphatidylinositol (GPI)-anchor biosynthesis | 10 | 3 | 7 |
| ko03250 | Viral life cycle - HIV-1 | 13 | 7 | 6 |
| ko00565 | Ether lipid metabolism | 9 | 6 | 3 |
| ko03440 | Homologous recombination | 21 | 9 | 12 |
| ko01250 | Biosynthesis of nucleotide sugars | 33 | 19 | 14 |
| ko00232 | Caffeine metabolism | 1 | 0 | 1 |
| ko00340 | Histidine metabolism | 6 | 3 | 3 |
| ko04130 | SNARE interactions in vesicular transport | 15 | 7 | 8 |
| ko00770 | Pantothenate and CoA biosynthesis | 13 | 7 | 6 |
| ko01240 | Biosynthesis of cofactors | 95 | 43 | 52 |
| ko00650 | Butanoate metabolism | 7 | 6 | 1 |
| ko00600 | Sphingolipid metabolism | 16 | 4 | 12 |
| ko00710 | Carbon fixation in photosynthetic organisms | 29 | 19 | 10 |
| ko04070 | Phosphatidylinositol signaling system | 25 | 15 | 10 |
| ko00601 | Glycosphingolipid biosynthesis - lacto and neolacto series | 1 | 1 | 0 |
| ko00410 | beta-Alanine metabolism | 19 | 13 | 6 |
| ko00960 | Tropane, piperidine and pyridine alkaloid biosynthesis | 10 | 2 | 8 |
| ko00660 | C5-Branched dibasic acid metabolism | 2 | 1 | 1 |
| ko00195 | Photosynthesis | 17 | 14 | 3 |
| ko00944 | Flavone and flavonol biosynthesis | 7 | 4 | 3 |
| ko03030 | DNA replication | 18 | 8 | 10 |
| ko00950 | Isoquinoline alkaloid biosynthesis | 8 | 1 | 7 |
| ko00250 | Alanine, aspartate and glutamate metabolism | 18 | 10 | 8 |
| ko00220 | Arginine biosynthesis | 14 | 4 | 10 |
| ko00290 | Valine, leucine and isoleucine biosynthesis | 5 | 3 | 2 |
| ko04122 | Sulfur relay system | 2 | 1 | 1 |
| ko03010 | Ribosome | 114 | 13 | 101 |
| ko00270 | Cysteine and methionine metabolism | 39 | 19 | 20 |
| ko00030 | Pentose phosphate pathway | 17 | 8 | 9 |
| ko00965 | Betalain biosynthesis | 1 | 1 | 0 |
| ko03420 | Nucleotide excision repair | 25 | 13 | 12 |
| ko00450 | Selenocompound metabolism | 5 | 4 | 1 |
| ko01230 | Biosynthesis of amino acids | 81 | 37 | 44 |
| ko00460 | Cyanoamino acid metabolism | 26 | 12 | 14 |
| ko00590 | Arachidonic acid metabolism | 3 | 2 | 1 |
| ko00196 | Photosynthesis - antenna proteins | 4 | 4 | 0 |
| ko00260 | Glycine, serine and threonine metabolism | 21 | 9 | 12 |
| ko03022 | Basal transcription factors | 16 | 6 | 10 |
| ko00440 | Phosphonate and phosphinate metabolism | 1 | 1 | 0 |
| ko00073 | Cutin, suberine and wax biosynthesis | 15 | 5 | 10 |
| ko00966 | Glucosinolate biosynthesis | 3 | 2 | 1 |
| ko01212 | Fatty acid metabolism | 23 | 12 | 11 |
| ko00100 | Steroid biosynthesis | 16 | 12 | 4 |
| ko00230 | Purine metabolism | 29 | 18 | 11 |
| ko00330 | Arginine and proline metabolism | 20 | 12 | 8 |
| ko00280 | Valine, leucine and isoleucine degradation | 17 | 9 | 8 |
| ko00300 | Lysine biosynthesis | 3 | 2 | 1 |
| ko00500 | Starch and sucrose metabolism | 74 | 52 | 22 |
| ko00900 | Terpenoid backbone biosynthesis | 14 | 9 | 5 |
| ko00470 | D-Amino acid metabolism | 1 | 0 | 1 |
| ko00240 | Pyrimidine metabolism | 17 | 8 | 9 |
| ko00785 | Lipoic acid metabolism | 9 | 3 | 6 |
| ko01210 | 2-Oxocarboxylic acid metabolism | 16 | 8 | 8 |
| ko01232 | Nucleotide metabolism | 22 | 10 | 12 |
| ko00760 | Nicotinate and nicotinamide metabolism | 4 | 4 | 0 |
| ko01100 | Metabolic pathways | 1055 | 512 | 543 |
| ko00730 | Thiamine metabolism | 5 | 3 | 2 |
| ko00040 | Pentose and glucuronate interconversions | 48 | 24 | 24 |
| ko04136 | Autophagy - other | 12 | 2 | 10 |
| ko00062 | Fatty acid elongation | 8 | 4 | 4 |
| ko03040 | Spliceosome | 88 | 34 | 54 |
| ko00640 | Propanoate metabolism | 10 | 6 | 4 |
| ko00670 | One carbon pool by folate | 3 | 2 | 1 |
| ko00514 | Other types of O-glycan biosynthesis | 2 | 2 | 0 |
| ko04144 | Endocytosis | 64 | 27 | 37 |
| ko00020 | Citrate cycle (TCA cycle) | 16 | 8 | 8 |
| ko00510 | N-Glycan biosynthesis | 11 | 3 | 8 |
| ko00630 | Glyoxylate and dicarboxylate metabolism | 17 | 8 | 9 |
| ko00970 | Aminoacyl-tRNA biosynthesis | 19 | 18 | 1 |
| ko01200 | Carbon metabolism | 88 | 49 | 39 |
| ko03050 | Proteasome | 13 | 5 | 8 |
| ko04712 | Circadian rhythm - plant | 25 | 17 | 8 |
| ko01040 | Biosynthesis of unsaturated fatty acids | 4 | 3 | 1 |
| ko03008 | Ribosome biogenesis in eukaryotes | 19 | 4 | 15 |
| ko03020 | RNA polymerase | 10 | 3 | 7 |
| ko04145 | Phagosome | 16 | 5 | 11 |
| ko03018 | RNA degradation | 34 | 15 | 19 |
| ko03083 | Polycomb repressive complex | 20 | 10 | 10 |
| ko04146 | Peroxisome | 29 | 14 | 15 |
| ko04120 | Ubiquitin mediated proteolysis | 41 | 17 | 24 |
| ko04141 | Protein processing in endoplasmic reticulum | 78 | 18 | 60 |
| ko00513 | Various types of N-glycan biosynthesis | 4 | 0 | 4 |
| ko04814 | Motor proteins | 24 | 11 | 13 |
| ko03013 | Nucleocytoplasmic transport | 29 | 17 | 12 |
| ko03082 | ATP-dependent chromatin remodeling | 14 | 10 | 4 |
| ko03015 | mRNA surveillance pathway | 29 | 24 | 5 |

**Table S8** Genes associated with root development and metabolic pathways in which these genes are involved

| ID | KEGG pathway |
| --- | --- |
| MD02G1071000 | ko04016: MAPK signaling pathway - plant |
| MD02G1267200 | ko04016: MAPK signaling pathway - plant |
| MD05G1243600 | ko04016: MAPK signaling pathway - plant |
| MD02G1013000 | ko04016: MAPK signaling pathway - plant; ko04075: Plant hormone signal transduction |
| MD05G1229400 | ko04016: MAPK signaling pathway - plant; ko04075: Plant hormone signal transduction |
| MD02G1149900 | ko04016: MAPK signaling pathway - plant; ko04075: Plant hormone signal transduction; ko04626: Plant-pathogen interaction |
| MD04G1238200 | ko04016: MAPK signaling pathway - plant; ko04075: Plant hormone signal transduction; ko04626: Plant-pathogen interaction |
| MD04G1238300 | ko04016: MAPK signaling pathway - plant; ko04075: Plant hormone signal transduction; ko04626: Plant-pathogen interaction |
| MD04G1238400 | ko04016: MAPK signaling pathway - plant; ko04075: Plant hormone signal transduction; ko04626: Plant-pathogen interaction |
| MD02G1097900 | ko04016: MAPK signaling pathway - plant; ko04626: Plant-pathogen interaction |
| MD00G1016500 | ko04075: Plant hormone signal transduction |
| MD02G1007200 | ko04626: Plant-pathogen interaction |
| MD02G1067500 | ko04626: Plant-pathogen interaction |
| MD03G1062400 | ko04626: Plant-pathogen interaction |
| MD03G1063500 | ko04626: Plant-pathogen interaction |
| MD03G1063600 | ko04626: Plant-pathogen interaction |
| MD04G1237900 | ko04626: Plant-pathogen interaction |
| MD04G1238100 | ko04626: Plant-pathogen interaction |
| MD04G1243600 | ko04626: Plant-pathogen interaction |
| MD06G1100500 | ko04626: Plant-pathogen interaction |
| MD09G1069300 | ko04626: Plant-pathogen interaction |
| MD09G1069400 | ko04626: Plant-pathogen interaction |


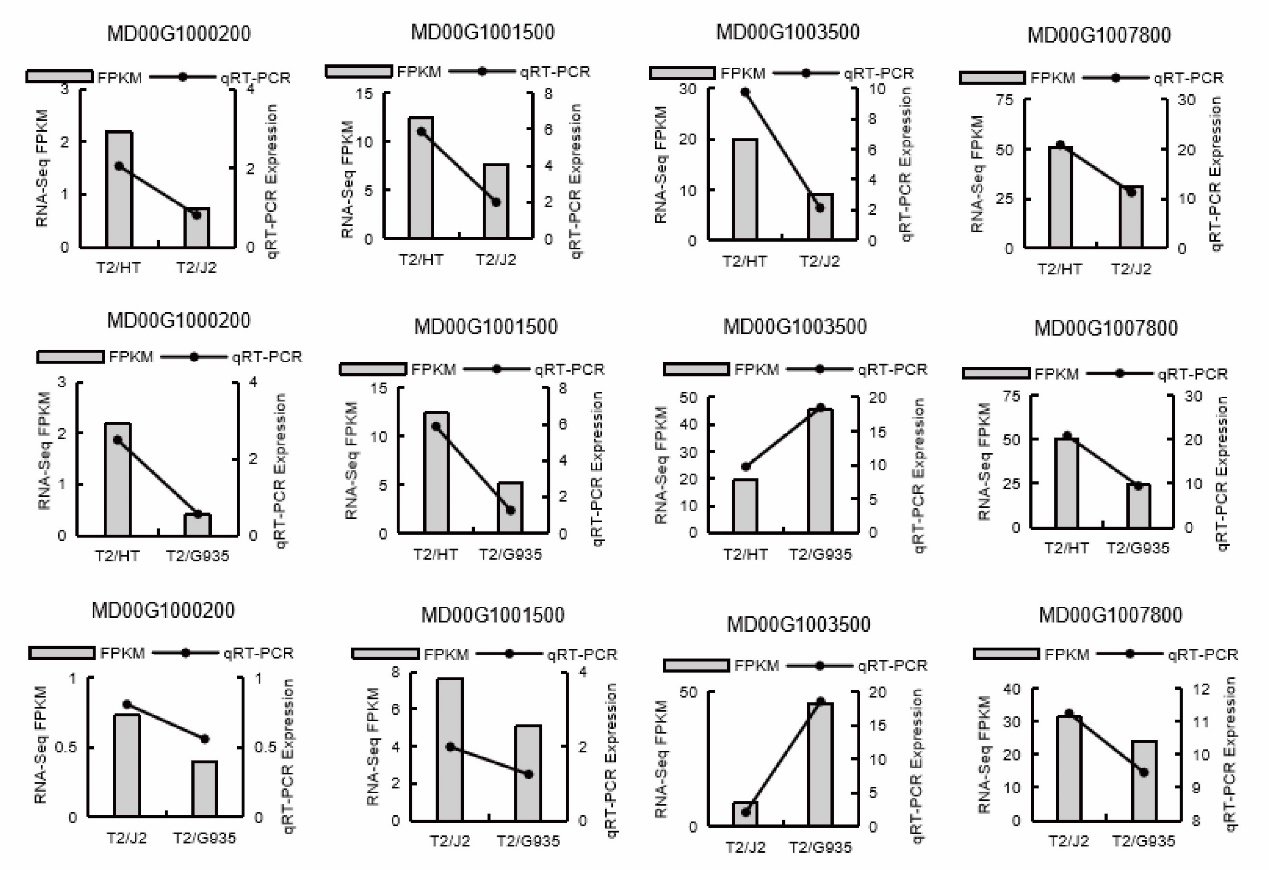


Fig. S1 qRT-PCR verification of DEGs, where gray bars represent RNA-seq data (log_2_FoldChange) and black dot represent qRT-PCR data (log_10_2^−∆∆CT^).
